# Establishment and characterization of hypomethylating agent-resistant cell lines, MOLM/AZA-1 and MOLM/DEC-5

**DOI:** 10.18632/oncotarget.14342

**Published:** 2016-12-28

**Authors:** Eun-Hye Hur, Seung-Hyun Jung, Bon-Kwan Goo, Juhyun Moon, Yunsuk Choi, Dae Ro Choi, Yeun-Jun Chung, Je-Hwan Lee

**Affiliations:** ^1^ Department of Hematology, Asan Medical Center, University of Ulsan College of Medicine, Seoul, Korea; ^2^ Cancer Evolution Research Center, College of Medicine, The Catholic University of Korea; ^3^ Integrated Research Center for Genome Polymorphism, College of Medicine, The Catholic University of Korea; ^4^ Division of Hematology and Hematological Malignancies, Ulsan University Hospital, University of Ulsan College of Medicine, Ulsan, Korea; ^5^ Division of Hemato-Oncology, Hallym University Chuncheon Sacred Heart Hospital, Hallym University College of Medicine, Chuncheon, Korea; ^6^ Department of Microbiology, College of Medicine, The Catholic University of Korea

**Keywords:** azacitidine, decitabine, MOLM-13, resistance, cytogenetics

## Abstract

Two hypomethylating agents (HMAs), azacitidine and decitabine, have demonstrated clinical activities in myelodysplastic syndrome (MDS) and acute myeloid leukemia (AML); however, potential problems include development of acquired resistance. HMA-resistant patients have very poor prognosis and this cohort of patients constitutes an important area of research. To understand the mechanisms underlying HMA-resistance and to overcome it, we established an azacitidine-resistant cell line, MOLM/AZA-1 and a decitabine-resistant cell line, MOLM/DEC-5 using MOLM-13. For cytogenetic characterization, we performed microarray-based comparative genomic hybridization (array-CGH), which identified a total of 15 copy number alterations (CNAs). Among these CNAs, eight regions in HMA-resistant cell lines showed CNA patterns distinct from the parental MOLM-13 genome. Single nucleotide polymorphism (SNP) microarray was also performed to obtain a more reliable interpretation of the identified CNAs, and all HMA-resistance-specific CNAs except one detected by array-CGH were successfully validated. In addition to CNAs, copy neutral loss of heterozygosity and mosaic loss events were identified in HMA-resistant cell lines. In our resistant cell lines, MDR-1 was not overexpressed, while DNMT3b was upregulated. Azacitidine and decitabine did not inhibit DNMT1, DNMT3a, or DNMT3b in both HMA-resistant cell lines, while they inhibited the enzymes in parental MOLM-13. We also developed mouse xenograft models using MOLM/AZA-1 and MOLM/DEC-5. Our *in vitro* and *in vivo* models of HMA-resistant cell lines will provide clues for the elucidation of molecular mechanisms related to the development of resistance to HMA and tools for the application of novel therapeutics for AML and MDS.

## INTRODUCTION

Post-mitotic modification of DNA methylation constitutes a major epigenetic regulatory mechanism for inactivating gene expression. The mechanisms controlling methylation are frequently dysregulated in cancer and aberrant methylation has been implicated in the pathogenesis of several hematologic malignancies, including acute myeloid leukemia (AML) and myelodysplastic syndrome (MDS) [1]. Currently, two hypomethylating agents (HMAs), azacitidine and decitabine, have been approved for the treatment of AML in older patients and MDS because clinical trials have shown that treatment with HMAs is effective and can improve clinical outcome in patients with these diseases [2–5]. However, a significant proportion of patients experience primary or secondary resistance to HMAs because approximately half the patients respond to these agents, and many of these patients subsequently experience disease progression while receiving treatment with HMAs. Prognosis after failure with HMAs is very poor in MDS as well as in AML [6, 7]; however, the mechanisms of resistance to HMAs are not fully understood [8].

Drug-resistant cell line models can be useful *in vitro* tools for assessing anticancer drug resistance in the clinical scenario. Cell line models with acquired resistance to anticancer drugs provide us with valuable information in elucidating the mechanisms underlying clinical anticancer drug resistance. Differential phenotypic and/or molecular changes between a drug-resistant cell line and its drug-sensitive counterpart can also suggest a locus of drug action inferred by the presence of those particular alterations [9]. In this study, we developed two HMA-resistant cell lines that show *in vitro* resistance to clinical doses of azacitidine and decitabine, respectively, from a human monocytic leukemia cell line, MOLM-13. The parental MOLM-13 cell line was established from the peripheral blood of a patient at relapse of AML, which had evolved from MDS, and the cell line had features of both AML and MDS [10]. We compared the cytogenetic and phenotypic features of parental and HMA-resistant MOLM-13 cell lines to provide the platforms for the clarification of HMA resistance mechanisms and we also developed HMA-resistant xenograft models using HMA-resistant MOLM-13 cell lines for the application of novel therapeutics for AML and MDS.

## RESULTS

### Establishment of HMA-resistant cell lines, MOLM/AZA-1 and MOLM/DEC-5

**Figure 1:**
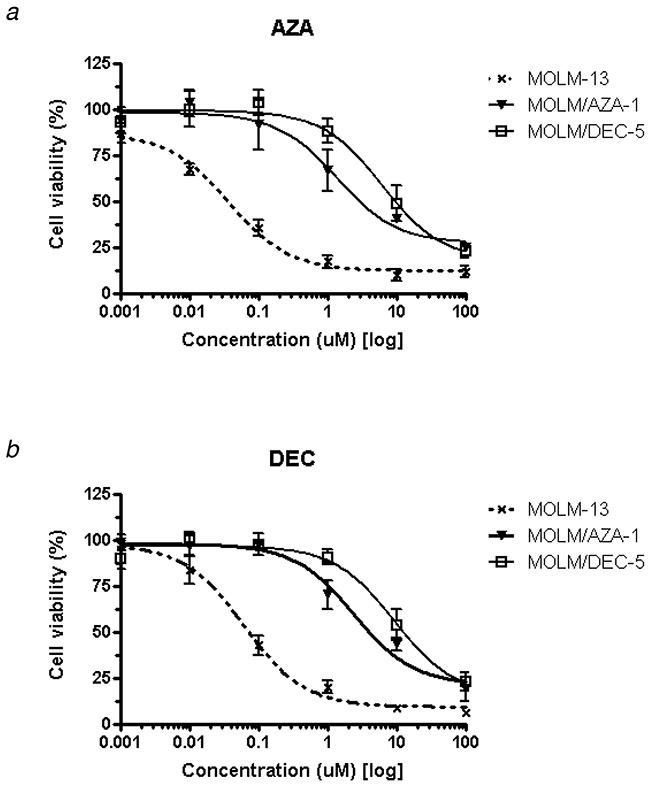
Establishment of hypomethylating agent-resistant cell lines using MOLM-13 The parental MOLM-13 cell line was sensitive to both azacitidine and decitabine. MOLM-13 cells were exposed continuously to increasing concentrations of azacitidine or decitabine, and we established an azacitidine-resistant cell line (MOLM/AZA-1) and a decitabine-resistant cell line (MOLM/DEC-5). The cell viability and proliferation was assessed by the luminescence-based CellTiter-Glo^®^ Luminescent Cell Viability Assay (Promega, Madison, WI). The concentrations of azacitidine or decitabine required for 50% growth inhibition were scored as IC_50_ values. **a**. IC_50_ value for azacitidine was 0.03804 μM in MOLM-13, 1.376 μM in MOLM/AZA-1, and 6.213 μM in MOLM/DEC-5. **b**. IC_50_ value for decitabine was 0.06294 μM in MOLM-13, 2.427 μM in MOLM/AZA-1, and 9.242 μM in MOLM/DEC-5.

We successfully established both azacitidine-resistant (MOLM/AZA-1) and decitabine-resistant (MOLM/DEC-5) cell lines from the parental cell line, MOLM-13. The IC_50_ values for azacitidine and decitabine in MOLM-13 were 0.03804 μM and 0.06294 μM, respectively, while the IC_50_ value for azacitidine in MOLM/AZA-1 was 1.376 μM (36-fold increase compared to the parental cell line) and that for decitabine in MOLM/DEC-5 was 9.242 μM (147-fold increase compared to the parental cell line) (Figure 1, Table 1). Both resistant cell lines showed cross-resistance to the other agent: IC_50_ value for azacitidine in MOLM/DEC-5 was 6.213 μM (163-fold increase compared to the parental cell line) and that for decitabine in MOLM/AZA-1 was 2.427 μM (39-fold increase compared to the parental cell line) (Figure 1, Table 1). We confirmed that the short tandem repeat profiles were matched between the parental MOLM-13 cell line and the two resistant cell lines (Supplementary Table 1).

**Table 1:** The IC_50_ of hypomethylating agents (HMAs) in MOLM-13 and MOLM-13-derived HMA-resistant cell lines (MOLM/AZA-1 and MOLM/DEC-5)

IC50 (μM)	Azacitidine	Decitabine
MOLM-13	0.03804	0.06294
MOLM/AZA-1	1.376	2.427
MOLM/DEC-5	6.213	9.242

### Copy number alterations specifically identified in HMA-resistant cell lines

**Figure 2:**
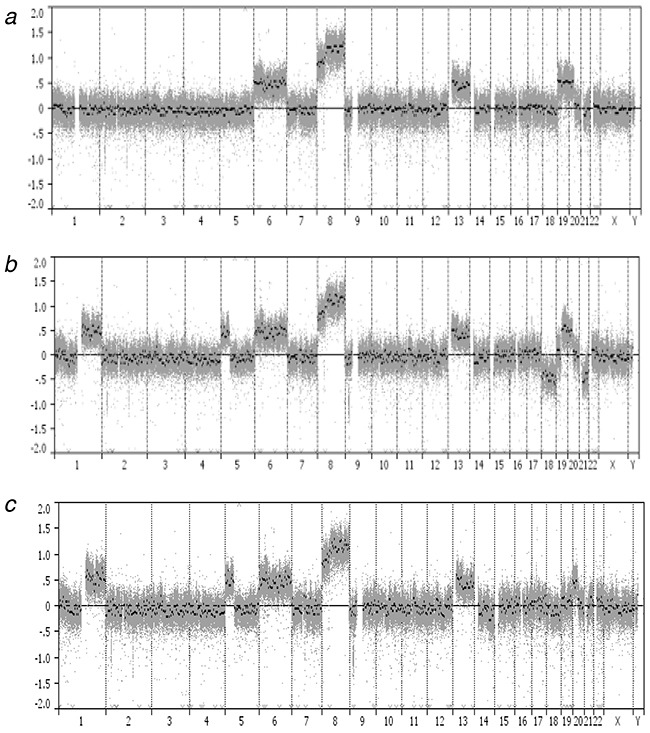
Genome-wide profiles of chromosomal alterations The *x*-axis represents individual chromosomes and the *y*-axis represents signal intensity ratio (each cell line genome/pooled normal genome) on a log_2_ scale. **a**. MOLM-13, **b**. MOLM/AZA-1, **c**. MOLM/DEC-5.

We performed microarray-based copy number profiling of parental and HMA-resistant MOLM-13 cell lines with pooled normal genomes as reference. The eight copy number gain on 6p, 6q, 8p, 8q, 13q, 19p, 19q, and 20p, and the one copy number loss on 9p21.3 were identified in the parental MOLM-13 genome (Figure 2a, Table 2). The 13 and 10 copy number alterations (CNAs) were also identified in the MOLM/AZA-1 and MOLM/DEC-5 genomes, respectively (Figure 2b, 2c, Table 2). Of note, eight regions showed distinct CNA patterns compared with the parental MOLM-13 genome. Copy number gains on 1q21.1–q44 and 5p15.33-p11 were identified in both MOLM/AZA-1 and MOLM/DEC-5. Copy number losses on 18p11.32-p11.21, 18q11.1-q23, and 21q11.2-q22.3 were detected only in the MOLM/AZA-1 genome, whereas copy number loss on 14q24.2–q32.33 was detected only in the MOLM/DEC-5 genome. Copy number gains on 19p13.3-p12 and 19q11-q13.43 detected in MOLM-13 were not identified in both resistance cell lines, suggesting their relative copy losses.

**Table 2:** Copy number alterations in MOLM-13, MOLM/AZA-1, and MOLM/DEC-5 genomes

Chromosome Region	Samples	Event	Cytoband	Cancer Gene Census*
chr1:144,009,907-249,250,621	MOLM/AZA-1, MOLM/DEC-5	Gain	q21.1–q44	*PDE4DIP, BCL9, ARNT, TPM3, MUC1, PRCC, NTRK1, SDHC, FCGR2B, PBX1, ABL2, TPR, MDM4, ELK4, SLC45A3, H3F3A, FH*
chr5:0-46,100,367	MOLM/AZA-1, MOLM/DEC-5	Gain	p15.33–p11	*IL7R, LIFR*
chr6:0-58,686,125	MOLM-13, MOLM/AZA-1, MOLM/DEC-5	Gain	p25.3–p11.2	*IRF4, DEK, HIST1H4I, TRIM27, POU5F1, DAXX, HMGA1, FANCE, PIM1, TFEB, CCND3*
chr6:61,000,000-171,115,067	MOLM-13, MOLM/AZA-1, MOLM/DEC-5	Gain	q11.1–q27	*PRDM1, ROS1, GOPC, STL, MYB, TNFAIP3, ECT2L, EZR, FGFR1OP, MLLT4*
chr8:0-43,396,776	MOLM-13, MOLM/AZA-1, MOLM/DEC-5	Gain	p23.3–p11.1	*PCM1, WRN, WHSC1L1, FGFR1, HOOK3*
chr8:46,943,457-146,364,022	MOLM-13, MOLM/AZA-1, MOLM/DEC-5	Gain	q11.1–q24.3	*TCEA1, PLAG1, CHCHD7, NCOA2, HEY1, COX6C, EXT1, MYC, NDRG1, RECQL4*
chr9:20,414,808-21,925,193	MOLM-13, MOLM/AZA-1, MOLM/DEC-5	Loss	p21.3	*MLLT3*
chr13:19,296,544-115,169,878	MOLM-13, MOLM/AZA-1, MOLM/DEC-5	Gain	q11–q34	*CDX2, FLT3, BRCA2, LHFP, LCP1, RB1, ERCC5*
chr14:72,033,418-107,349,540	MOLM/DEC-5	Loss	q24.2–q32.33	*TSHR, TRIP11, GOLGA5, DICER1, TCL6, TCL1A, BCL11B, AKT1, IGH@*
chr18:0-14,966,054	MOLM/AZA-1	Loss	p11.32–p11.21	
chr18:18,529,851-78,077,248	MOLM/AZA-1	Loss	q11.1–q23	*ZNF521, SS18, MALT1, BCL2*
chr19:0-24,340,741	MOLM-13	Gain	p13.3–p12	*FSTL3, STK11, TCF3, GNA11, SH3GL1, MLLT1, DNM2, SMARCA4, LYL1, BRD4, TPM4, JAK3, ELL*
chr19:28,272,497-59,042,827	MOLM-13, MOLM/AZA-1	Gain	q11–q13.43	*CCNE1, CEBPA, AKT2, CD79A, CIC, BCL3, CBLC, ERCC2, KLK2, PPP2R1A, ZNF331, TFPT*
chr20:0-25,904,169	MOLM-13, MOLM/AZA-1, MOLM/DEC-5	Gain	p13–p11.1	
chr21:14,420,615-48,129,895	MOLM/AZA-1	Loss	q11.2–q22.3	*OLIG2, RUNX1, ERG, TMPRSS2, U2AF1*

* http://cancer.sanger.ac.uk/census/

To validate the identified CNAs, we repeated the array-comparative genomic hybridization (array-CGH) for the MOLM/AZA-1 and MOLM/DEC-5 cell lines with genomic DNA from the parental MOLM-13 cell line as a reference for each hybridization. All the CNAs identified in these array-CGH analyses can be interpreted to be acquired during development of HMA-resistance. Through this validation, all of the HMA-resistance-specific CNAs were successfully validated (Supplementary Figure 1). In particular, 24-Mb-sized regions on 19p where *DNMT1*gene resides showed copy number gain in the parental MOLM-13 genome, but not in the MOLM/AZA-1 and MOLM/DEC-5 genomes. A 31-Mb-sized region on 19q in the MOLM/DEC-5 genome showed relative copy loss, while the MOLM/AZA-1 genome showed the same copy number as the parental MOLM-13 genome.

### Comparison of single nucleotide polymorphism (SNP) microarray and array-CGH results

**Figure 3:**
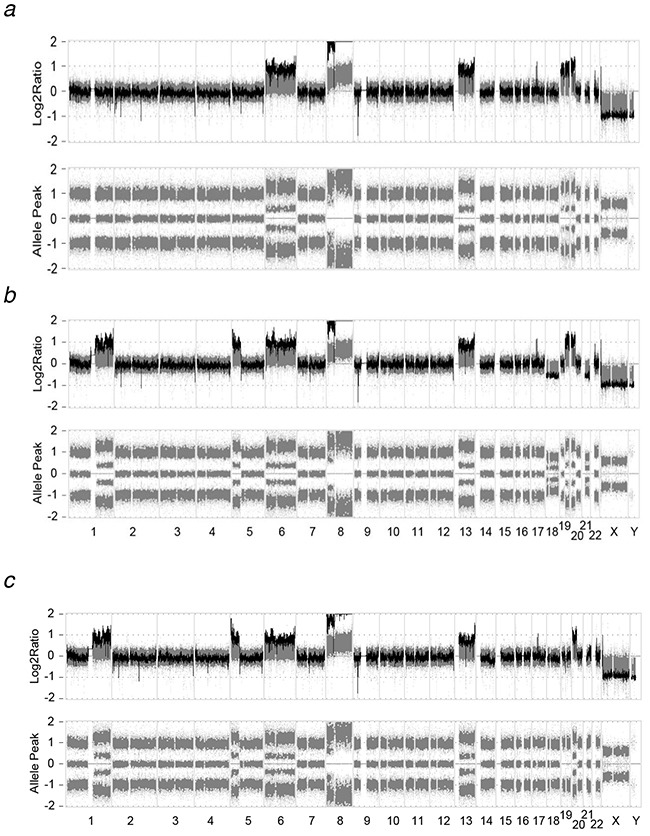
The log_2_ ratio of array-CGH and allele peak view of SNP microarray The CNAs detected by array-CGH were supported by the allele peak plots by SNP microarray. For example, a 135-Mb-sized region of copy number gain on chromosome 1 identified by array-CGH in MOLM/AZA-1 and MOLM/DEC-5 was also detected by SNP microarray and clearly showed four heterozygous clusters of SNP markers in the allele peak plot. **a**. MOLM-13, **b**. MOLM/AZA-1, **c**. MOLM/DEC-5.

To obtain a more reliable interpretation, we performed whole-genome SNP microarray for the genomes of parental MOLM-13 and HMA-resistant cell lines. Through the SNP microarray, all HMA-resistance-specific CNAs except one (14q24.2–q32.33 in MOLM/DEC-5) detected by array-CGH were successfully validated (Supplementary Figure 1). In addition, allele peak plots offered further support for the copy number alterations. For example, a 135-Mb-sized region of copy number gain on chromosome 1 identified by array-CGH in MOLM/AZA-1 and MOLM/DEC-5 was also detected by SNP microarray and clearly showed four heterozygous clusters of SNP markers in the allele peak plot, while no copy number gain or heterozygous clusters of SNP markers in the allele peak plot, while no copy number gain or heterozygous clusters of SNP markers were detected in the MOLM-13 genome (Figure 3). Likewise, copy number loss on chromosome 9 identified by array-CGH in HMA-resistant cell lines clearly showed two heterozygous clusters of SNP markers in the allele peak plot (Supplementary Figure 2).

Next, we compared the HMA-resistance-specific CNAs to those identified in representative myeloid neoplasm cell lines. For the comparison, we downloaded the raw SNP microarray data of three AML cell lines (HL-60, SKM-1 and U-937) from Gene Expression Omnibus database (GSE36138) and identified CNAs with the same detection criteria used in this study. Of the three cell lines, two (SKM-1 and U-937) exhibited a copy gain on 1q which was identified as HMA-resistance-specific CNA in this study (Supplementary Figure 3). In addition to the CNAs, we identified a copy-neutral LOH and mosaic loss event in HMA-resistant cell lines. The 19p13.3–p12 region, which showed copy number gain in MOLM-13, was copy neutral in both HMA-resistant cell lines, and LOH was identified only in MOLM/DEC-5 (Figure 3 and Supplementary Figure 4a). The 31-Mb-sized copy-neutral LOH was also identified at the distal end of chromosome 19 in MOLM/DEC-5, but not in the other cell lines. The loss of entire chromosomes 18 and 21q was identified only in MOLM/AZA-1 and showed four heterozygous clusters of SNPs in allele peak, revealing a mosaic loss event (Supplementary Figure 4b). Taken together, these results suggest that MOLM/AZA-1 and MOLM/DEC-5 might each acquire HMA-resistance through a different mechanistic basis.

### Expression of cancer-related genes in the HMA-resistance-specific CNAs

**Figure 4:**
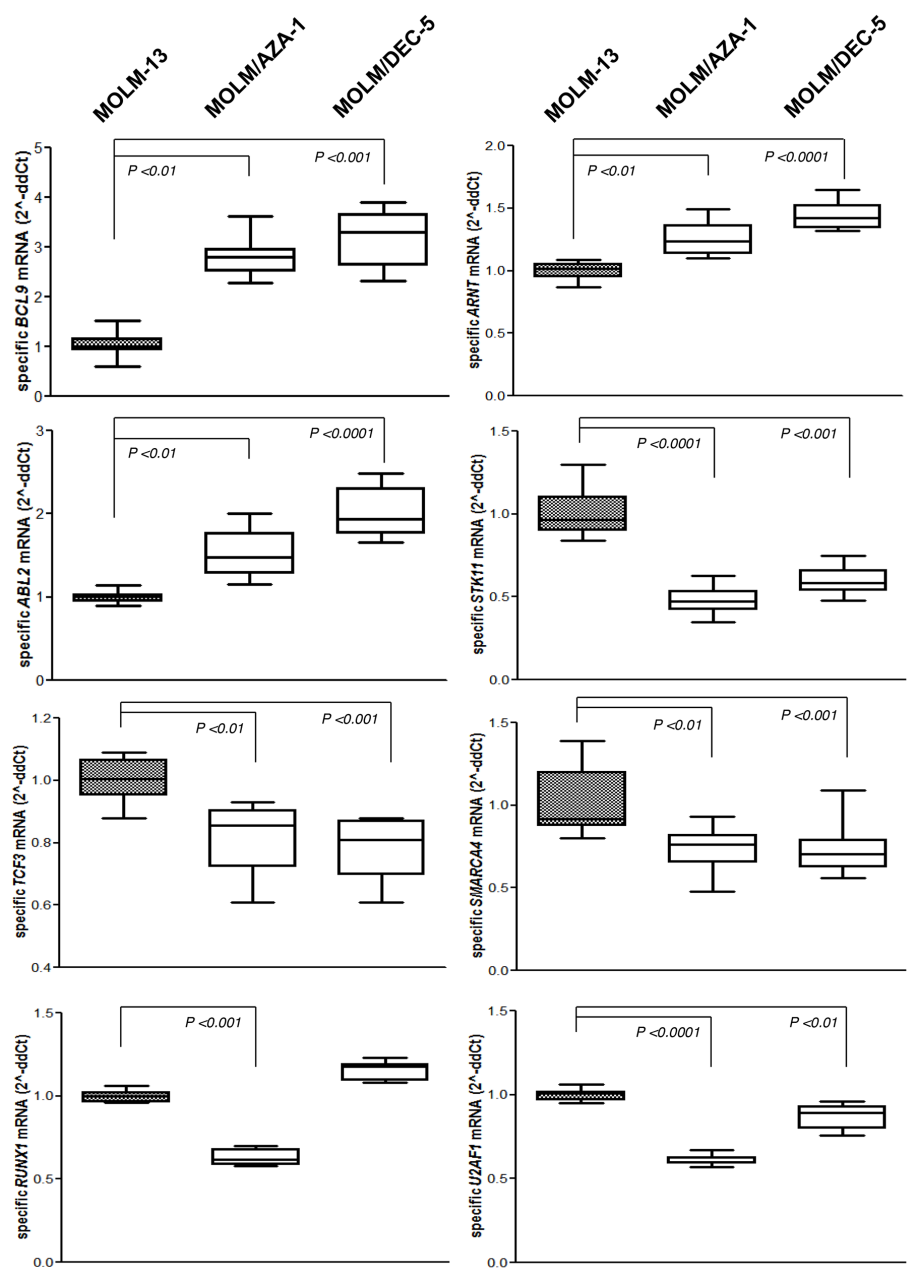
Expression profile of the cancer related genes Expression levels of 8 cancer-related genes in the HMA-resistance-specific CNAs were confirmed by real-time qPCR assay in the indicated group of cell lines. Relative mRNA expression levels of each gene were compared with MOLM-13 cell line. Standard errors of the statistical mean were indicated by the error bars. Mean values were obtained from triplicate data sets.

Several cancer-related genes such as *STK11, SMARCA4*, and *RUNX1* were located within the HMA-resistance-specific CNAs (Table 2). To investigate whether the HMA-resistance-specific CNAs influence gene expression, we assayed the expression of 8 cancer-related genes by quantitative reverse transcription PCR (RT-qPCR) including *BCL9*, *ARNT*, *ABL2, STK11, TCF3, SMARCA4, RUNX1*, and *U2AF1* (Figure 4). Expressions of three genes (*BCL9, ARNT*, and *ABL2*) in copy gain region on 1q21.1–q44 identified from both HMA-resistant cell lines were significantly higher in HMA-resistant cell lines compared to MOLM-13, whereas expressions of three genes (*STK11, TCF3*, and *SMARCA4*) in copy loss region on 19p13.3–p12 identified from both HMA-resistant cell lines were significantly lower in HMA-resistant cell lines compared to MOLM-13. Expressions of *RUNX1* and *U2AF1* genes in a copy number loss on 21q11.2–q22.3 identified from MOLM/AZA-1 were significantly lower in MOLM/AZA-1 compared to MOLM-13 and MOLM/DEC-5. To infer the biological implication of the cancer-related genes, we performed ontology/pathway analyses using the KEGG and DAVID tools [11] and we found that the cancer-related genes in HMA-resistance-specific CNAs were significantly associated with many tumorigenesis-related gene functions including ‘transcriptional misregulation in cancer’ (*P*=3.6×10^-11^), ‘cell differentiation’ (*P*=0.004), ‘PI3K-Akt signaling pathway’ (*P*=0.013), and ‘negative regulation of cell growth’ (*P*=0.023) (Supplementary Table 2). Taken together, HMA-resistance-specific CNAs may act to regulate multiple cancer-related genes in a dose-dependent manner.

### Phenotypic evaluation of HMA-resistant cell lines

**Figure 5:**
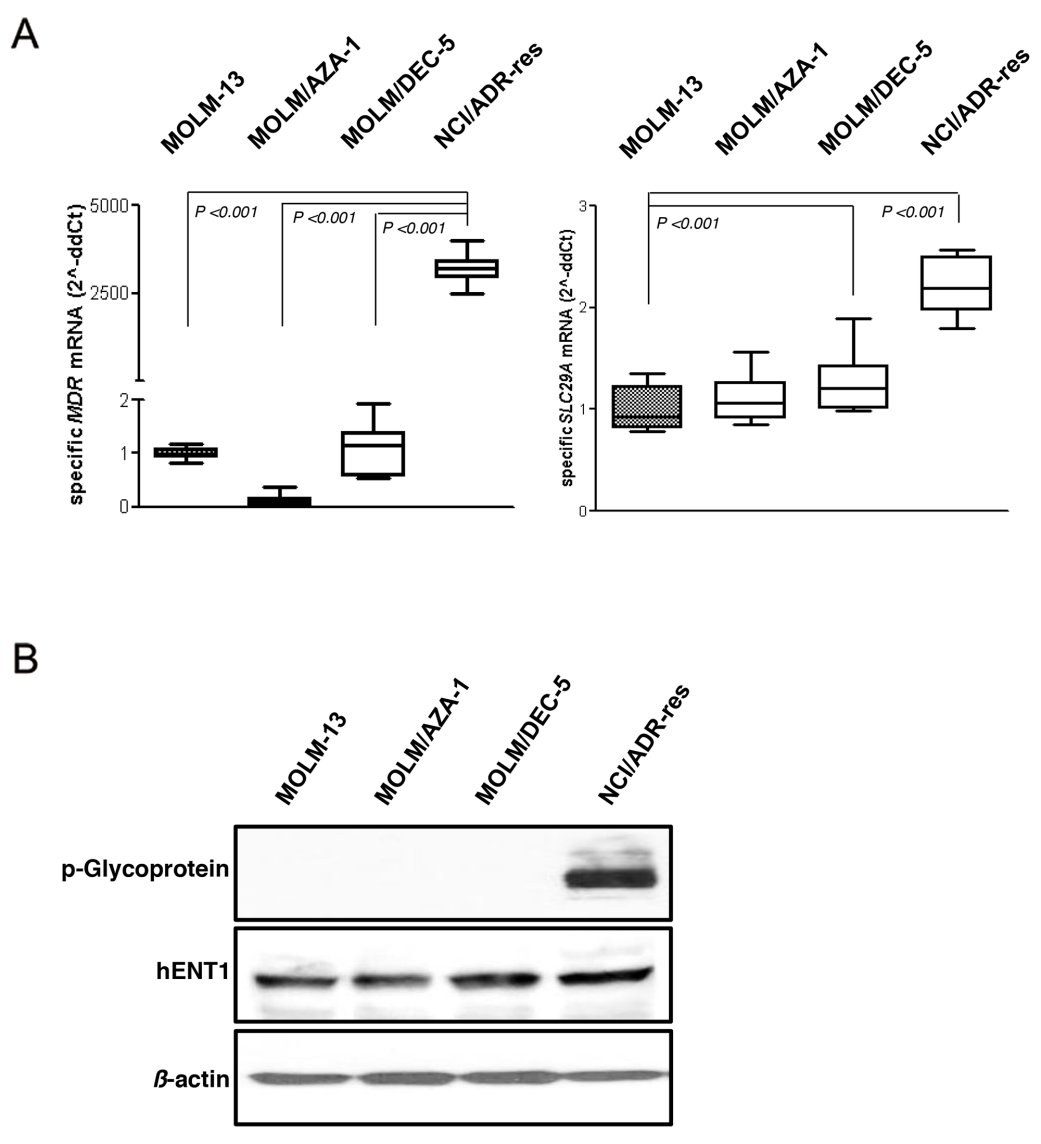
Assay for the *MDR1* gene expression **a**. mRNA expression of the *MDR1* and *SLC29A* gene using real-time quantitative polymerase chain reaction assay. Results are presented as the expression relative to *beta-actin* mRNA expression. Values are mean ± SEM for three independent experiments. **b**. Protein expression of P-glycoprotein and hENT1 using western blot assay. NCI/ADR-Res cells are used as a positive control for P-glycoprotein. *β*-actin was measured as a loading control.

We evaluated the expression of the resistant genes, P-glycoprotein (Pgp) and hENT1 in HMA-resistant cell lines (Figure 5). Pgp, encoded by the *MDR1* gene, is an ATP-dependent efflux pump with broad substrate specificity, and hENT1, encoded by the *SLC29A* gene, is an equilibrative nucleoside transporter family. As a positive control of *MDR1* and Pgp expression, we used an NCI/ADR-Res cell line. Expression level of *MDR1* gene in MOLM-13 (1.0 fold), MOLM/AZA-1 (0.1 fold) and MOLM/DEC-5 (1.1 fold) was significantly lower than those of NCI/ADR-Res (3241.9 fold). The protein expressions of Pgp reflected the gene expressions of the cell lines (Figure 5b). *SLC29A* gene expressions and hENT levels were not significantly different between MOLM-13 and HMA-resistant cell lines (Figure 5b). These results indicate that development of HMA resistance in our cell lines was not related to increase of Pgp or hENT1 activities.

**Figure 6:**
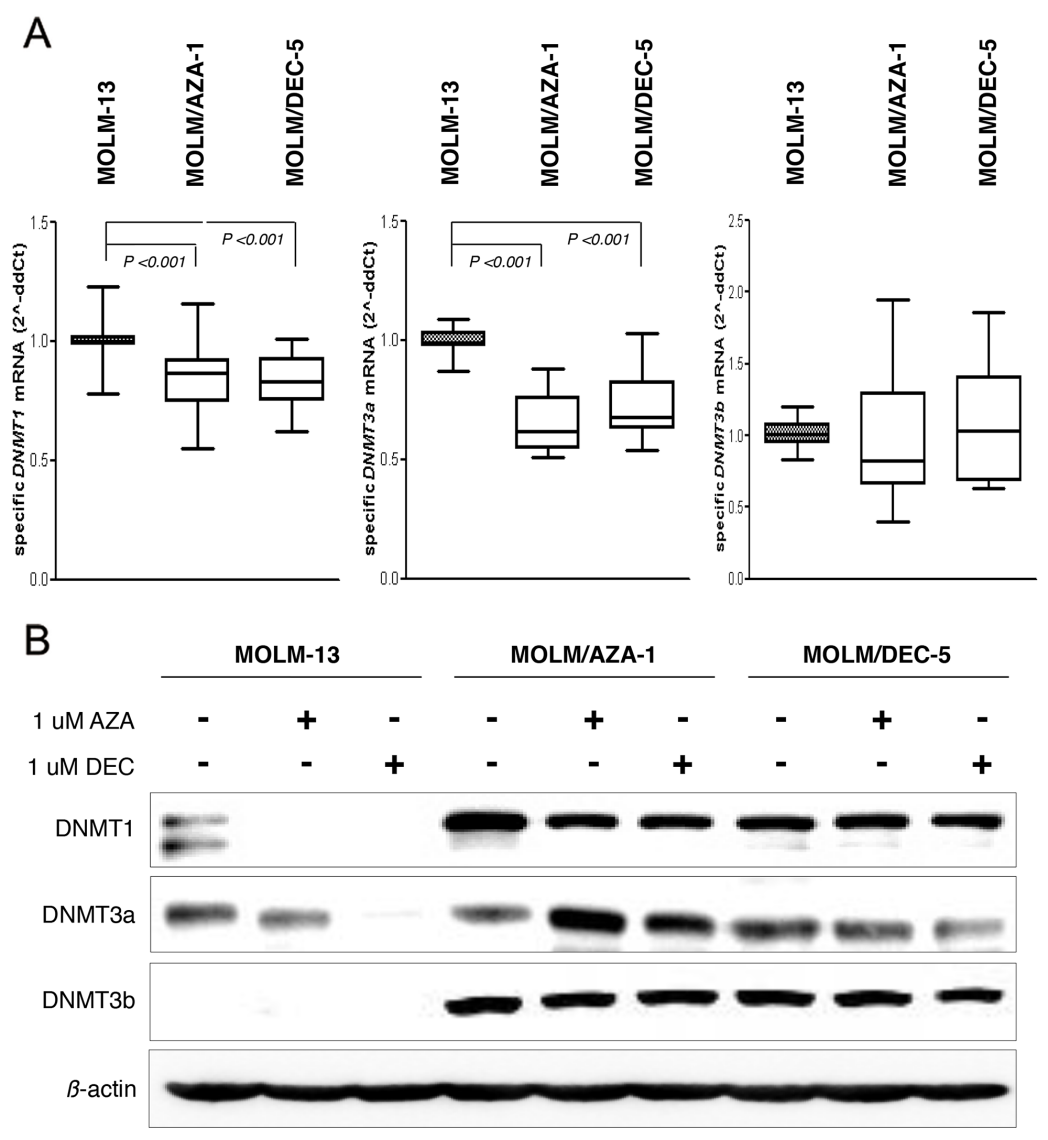
Expression of DNMT family proteins **a**. mRNA expression of the *DNMT1*, *DNMT3a*, and *DNMT3b* genes using real-time quantitative polymerase chain reaction assay. Results are presented as the expression relative to *beta-actin* mRNA expression. Values are mean ± SEM for three independent experiments. **b**. Protein expression of DNMT1, DNMT3a, and DNMT3b pre- and post-treatment with azacitidine or decitabine for 48 h. *β*-Actin was measured as a loading control.

We also assessed the expression of DNA methyltransferase (DNMT) family proteins; DNMT1, DNMT3a, and DNMT3b. RQ-PCR assay showed that mRNA expression of DNMT family genes in MOLM/AZA-1 and MOLM/DEC-5 slightly decreased compared to parental MOLM-13 (Figure 6a). Protein levels of DNMT1 and DNMT3a in MOLM/AZA-1 and MOLM/DEC-5 were equivalent compared to parental MOLM-13. DNMT3b was not detected in parental MOLM-13, but protein levels of DNMT3b significantly increased in MOLM/AZA-1 and MOLM/DEC-5 (Figure 6b). Treatment with azacitidine or decitabine decreased both DNMT1 and DNMT3a to undetectable levels in parental MOLM-13, whereas treatment with these agents did not significantly reduce the protein levels of DNMT1, DNMT3a, and DNMT3b in HMA-resistant cell lines (MOLM/AZA-1 and MOLM/DEC-5) (Figure 5b). R882, hot-spot locus of DNMT3a, is not mutated in all cell lines (Supplementary Figure 5).

### Establishment of HMA-resistant xenograft models

**Figure 7:**
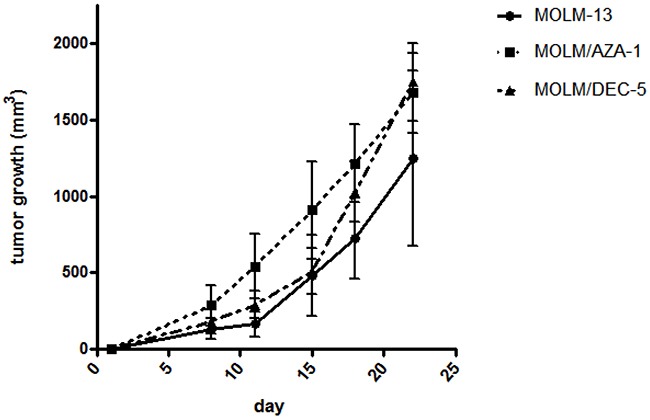
Establishment of HMA-resistant xenograft models The *x*-axis denotes days after subcutaneous injection of MOLM-13, MOLM/AZA-1, or MOLM/DEC-5 cells into Balb/c nu/nu mice (n=7 per cell line), and the *y*-axis denotes tumor volume. Tumor volume was monitored twice a week for 3 weeks. HMA-resistant cell lines showed more aggressive increases in tumor volume around 22 days compared to the parental MOLM-13 cell line. Data are shown as means ± SEM.

We exploited tumor growth of MOLM/AZA-1 and MOLM/DEC-5 cell lines to establish HMA-resistant xenograft models. The parental MOLM-13 and two HMA-resistant cell lines were subcutaneously transplanted into Balb/c nu/nu mice (n=21) to determine the establishment of *in vivo* tumor models. Interestingly, HMA-resistant cell lines showed more aggressive increases in tumor volume compared to the parental MOLM-13 cell line and tumor volumes of HMA-resistant cell lines were higher than that of MOLM-13 on 22 days (Figure 7). The finding might be related with the differences of *in vitro* doubling times between HMA-resistant cell lines and MOLM-13; 17 hours with MOLM/AZA-1 or MOLM/DEC-5 and 41.1 hours with MOLM-13.

## DISCUSSION

The clinical course after failure of treatment with HMA is usually dismal and HMA failure is an unmet clinical need in the management of MDS and elderly AML patients [6, 7]. Thus, it is important to evaluate the mechanism of HMA resistance and to overcome this resistance [8, 12]. For the investigations related to HMA resistance, we developed *in vitro* and *in vivo* HMA resistance models using the MOLM-13 cell line that has biologic features of both AML and MDS. We also characterized the phenotypic and cytogenetic properties of our HMA-resistant cell lines (MOLM/AZA-1 and MOLM/DEC-5). Overexpression of the *MDR1* gene was not associated with the development of HMA resistance in our cell line models because the mRNA and protein levels of the *MDR1* gene were not elevated in resistant cell lines compared to the parental cell line. Interestingly, protein levels of DNMT3b were upregulated in HMA-resistant cell lines while it was not expressed in the parental MOLM-13 cell line. Furthermore, DNMT enzymes (DNMT1, DNMT3a, and DNTM3b) were not effectively inhibited by DNMT inhibitors in HMA-resistant cell lines, although the DNMT enzymes (DNMT1 and DNMT3a) were effectively inhibited by both DNMT inhibitors (azacitidine and decitabine) in the parental cell line. DNMT1 is known to methylate CpG residue and to be responsible for maintaining methylation patterns established during development [13], while DNMT3a and DNMT3b are involved in *de novo* DNA methylation in newly synthesized DNA molecules [14]. However, DNMT3a and DNMT3b also carry out maintenance of DNA methylation, correcting the errors of DNMT1. Human cancer cells with DNMT1 knockout were found to retain their inherited methylation pattern, suggesting maintenance activity by the expressed DNMT3a and DNMT3b [15]. A recent study using an intestinal epithelium model also showed that DNMT3b could compensate for the DNMT1 deficiency [16]. DNMT3a and DNMT3b can bind to both unmethylated and hemimethylated DNA substrates and, hence, potentially play a role in both maintenance and *de novo* modifications. Thus, HMA appears to have to inhibit DNMT3a and DNMT3b as well as DNMT1 to exert anti-tumor activities. An *in vitro* study demonstrated that azacitidine and decitabine decreased protein expression of DNMT1 and DNMT3a in MDS-derived cell lines [17]. In our HMA-resistant cell line models, the fact that expression of DNMT enzymes (especially, DNMT3b) is elevated and the DNMT enzymes are not effectively suppressed with azacitidine or decitabine, seems to be associated with resistance to azacitidine and decitabine.

For cytogenetic characterization of our HMA-resistant cell lines, array-CGH and SNP microarray were performed. Eight regions showed distinct CNA patterns between parental MOLM-13 and one or both HMA-resistant cell lines (Table 2), and additional copy-neutral LOH events were identified (Figure 3). Some of the HMA-resistance-specific CNAs were shared by both resistant cell lines, while others were found in only one of two resistant cell lines. Our data also suggested that the mechanisms of acquired resistance might be different in MOLM/AZA-1 compared with MOLM/DEC-5, although both cell lines showed cross-resistance to both azacitidine and decitabine. It is well known that azacitidine and decitabine have different modes of action [18]. In previous studies, the two agents were differently metabolized, induced different effects on cell viability, and caused different sets of gene expression with little overlap [19, 20], while a substantial overlap of genes were demethylated by both agents [21]. The cytogenetic alterations in the HMA-resistant cell lines affected the expression of some cancer-related genes located in the resistance-specific CNA regions. Indeed, the genes were significantly associated with tumorigenesis-related pathways. Especially, ‘transcriptional misregulation in cancer’ and ‘PI3K-Akt signaling’ pathways have been reported to play a role in mediating drug resistance [22, 23]. Our data suggest that HMA-resistance-specific CNAs may play a role in mediating the resistance through gene expression regulation. However, we only assessed the expression levels of few cancer-related genes rather than global transcriptional profiling. Further investigation of gene expression with transcriptome sequencing will be needed to understand HMA-resistance mechanisms.

Drug-resistant cell lines are usually established by continuous exposure of drug-sensitive cell lines to graded concentrations of the drug. Azanucleoside drugs are generally considered to be unstable and half-life times were 7 h for azacitidine and 21 h for decitabine at physiologic media [18]. Thus, it is difficult to develop azacitidine- or decitabine-resistant cell lines. So far, three research groups have reported five azacitidine-resistant cell lines using U937, HL-60, THP-1, and SKM-1 (Table 3) [24–26]. Decitabine-resistant cell lines have not been reported, although decitabine-resistant cells derived from HL-60 were investigated [20]. We established both azacitidine- and decitabine-resistant cell lines from MOLM-13 that had features of both AML and MDS. MOLM-13 cells are sensitive to azacitidine or decitabine, and our resistant cell lines have much higher IC_50_ values compared to prior azacitidine-resistant cell lines: 36-fold (MOLM/AZA-1) and 147-fold (MOLM/DEC-5) higher IC_50_ than the parental cell line. We also developed *in vivo* xenograft models with HMA-resistant cell lines (Figure 6). Our *in vitro* and *in vivo* models for HMA-resistance could serve as valuable tools for the evaluation of novel agents or therapeutic interventions in AML and MDS. Our study contributes to better understanding of HMA-resistance in patients treated with HMAs. Considering that only half of patients respond to HMAs, it is also important to determine whether or not the same mechanisms are involved in the acquired and primary resistances to HMAs.

**Table 3:** Comparison of hypomethylating-agent-resistant cell lines

	This study	Imanishi et al. [25]	Sripayap et al. [26]	Cluzeau et al. [24, 27]
Parental cell line	MOLM-13	U937, HL-60	THP-1, HL-60	SKM-1
Hypomethylating agent (HMA)	Azacitidine, Dectabine	Azacitidine	Azacitidine	Azacitidine
Fold difference of IC_50_ compared to parental cell line	36 (azacitidine)147 (decitabine)		9.77 (THP-1) 6.73 (HL-60)	
MDR1 gene expression	No change	No change	No change	
Expression of DNMT enzymes	DNMT3b(↑) DNMT1/3a(→)	DNMT3a(↓) DNMT1(→)	DNMT1/3a/3b(→)	
Features of resistant cell lines	Several HMA-resistant specific copy number alterations and loss of heterozygosity	Downregulation of pyrimidine metabolism genes	BCL2L10(↑)	BCL2L10(↑) No SNP alterations The 15 MDS-associated gene mutation patterns were the same in both parental and resistant cell lines
Possible resistance mechanisms	Upregulation of DNMT3B Lack of inhibition of DNMT enzymes by HMAs	Activation of DNA damage response through ATM kinase	UCK2 gene mutation Lack of inhibition of DNMT enzymes by azacitidine	Upregulation of BCL2L10

DNMT, DNA methyltransferase; ATM, ataxia telangiectasia mutation

In conclusion, our HMA-resistant cell line models will provide clues for the elucidation of molecular mechanisms related to the development of resistance to HMA and tools for the application of novel therapeutics for AML and MDS.

## MATERIALS AND METHODS

### Cell line and cell proliferation assay

Molm-13 (DSMZ, Germany) cell line was cultured at 37°C in 5% CO_2_ in RPMI-1640 medium containing 10% (v/v) fetal bovine serum and1% penicillin/streptomycin (Invitrogen, Carlsbad, CA, USA).

Cell viability was assessed using the luminescence-based CellTiter-Glo^®^ Luminescent Cell Viability Assay (Promega, Madison, WI, USA) in accordance with the manufacturer's instructions. Briefly, cells were plated at 1,000–3,000 per well in a 96-well opaque plate and were incubated in complete growth medium. Cells were treated with various concentrations of azacitidine or decitabine, purchased from Sigma Aldrich (St Louis, MO, USA) and were prepared as a 50-mmol/L stock solutions in dimethyl sulfoxide (DMSO). After 48 h, cell viability was determined by measuring luminescent signals with a VICTOR^TM^ X Light luminescence plate reader (PerkinElmer, Waltham, MA, USA). The concentrations of azacitidine or decitabine required for 50% growth inhibition were scored as IC_50_ values.

### Establishment of HMA-resistant cell lines

The parental MOLM-13 cell line was sensitive to both azacitidine and decitabine *in vitro*. MOLM-13 cells were exposed continuously to gradually increasing concentrations of azacitidine (0.5 pM to 50 nM) and decitabine (0.05 nM to 1 μM), and the cells acquired resistance to azacitidine or decitabine. The resistant cells were isolated by a series of stepwise selections and two HMA-resistant cell lines, MOLM/AZA-1 and MOLM/DEC-5, were cloned by the limiting dilution method. The degree of resistance was calculated by dividing the IC_50_ value of the resistant cells by that of the parental cells.

### Authentication of MOLM/AZA-1 and MOLM/DEC-5 by analysis of short tandem repeats

To prove the derivation of MOLM/AZA-1 and MOLM/DEC-5 cell lines from the parental MOLM-13 cell line, short tandem repeat profiling of all three cell lines was performed and compared. Fifteen tetranucleotide repeat loci and the amelogenin gender-determining marker were amplified using AmpFℓSTR^®^ Identifiler^®^ Plus PCR Amplification Kit (Applied Biosystems, CA, USA) and analyzed by 3730 DNA analyzer and Peak scanner (Applied Biosystems, CA, USA).

### Array-CGH and data processing

DNA copy number profiling was performed using Agilent Sure Print G3 Human CGH Microarray 180K (Agilent Technologies, Santa Clara, CA, USA). Array-CGH experiments were conducted according to the manufacturer's instructions. In brief, 1 μg of each genomic DNA from MOLM-13, MOLM/AZA-1, and MOLM/DEC-5 cells was labeled with Cy5-dCTP (PerkinElmer, Waltham, MA) and reference DNA from normal individuals (Promega, Fitchburg, WI, USA) was labeled with Cy3-dCTP (PerkinElmer, Waltham, MA, USA). Labeled DNA was applied to the array with hybridization buffer and human Cot-1 DNA (HybMasker, ConnectaGen, Seoul, Korea). Array slides were incubated for 24 h at 65°C. After washing and scanning the arrays, images were analyzed with Feature Extraction Software v10.7.3.1 (Agilent Technologies, Santa Clara, CA, USA). Probe mapping was conducted according to its genomic location in the UCSC genome browser (Human NCBI37/hg19). The Rank Segmentation statistical algorithm in NEXUS software v7.5 (Biodiscovery Inc., El segundo, CA) was used to define copy number alterations in each sample.

### SNP microarray and data processing

Genome-wide SNP genotyping was conducted using the CytoScan^®^ HD microarray, which contains 743,304 SNP markers and 1,953,246 copy number markers (Affymetrix, Santa Clara, CA, USA), according to manufacturer's instructions. The average marker spacing is 1.1 kb. The signal intensity extraction and copy number alteration detection were conducted using Chromosome Analysis Suite (Affymetrix).

### RT-qPCR assay

Total RNAs were extracted using QIAzol Lysis Reagent (Qiagen, Valencia, CA, USA) and converted into cDNA using Revert Aid premium reverse transcriptase (Thermo scientific, South Logan, UT, USA) according to manufacturer's instructions. The mRNA transcript levels were quantified by RQ-PCR on LightCycler 96 (Roche, Indianapolis, IN) using the primer sets (Supplementary Table 3). After an initial denaturation step at 95°C for 5 min, amplification occurred over 45 cycles of denaturation at 95°C for 30 s, annealing at 60°C for 30 s, and extension at 72°C for 30 s. Each sample was tested in triplicate, and gene expression levels were normalized to those of *beta-actin*.

### Western blot analysis

The cells were lysed in lysis buffer (cell signaling tech. Beverly, MA, USA) for 15 min on ice. The protein concentration of the lysates was measured using a Bradford assay (Bio-Rad, Hercules, CA, USA). Protein extracts (50 μg) were separated by 6% sodium dodecyl sulfate-polyacrylamide gel electrophoresis and were transferred onto polyvinyl difluoride membranes. After blocking with 2% skim milk for 1 h, the membranes were incubated with primary antibodies overnight at 4°C, then with secondary antibody conjugated with horseradish peroxidase (Enzo Life Sciences, Farmingdale, NY, USA) for 2 h. The membrane was visualized by Super Signal^®^ West Pico Chemiluminescent Substrate (Thermo scientific, Rockford, IL, USA) and images were produced using medical X-ray film blue (Agfa health care NV, Belgium). Specific antibodies were as follows: p-glycoprotein antibody (Merck Millipore, Billerica, MA, USA), hENT1 (Novus Biologicals, Littleton, CO, USA), DNMT1 (R&D Systems, Minneapolis, MN, USA), DNMT3a (Abcam, Cambridge, MA, USA), DNMT3b (Abcam), and *β*-actin (Sigma, St. Louis, MO, USA).

### Establishment of HMA-resistant xenograft models

Each dose of 5 × 10^6^ cells of parent MOLM-13, MOLM/AZA-1, and MOLM/DEC-5 were mixed with matrigel (BD Bioscience, NJ, USA) and were injected subcutaneously through 22-gauge needles into the flank of 6-week-old BALB/c nu/nu mice (SLC, Inc. Japan) under isoflurane anesthesia. Mice were housed in a laminar flow caging system, and all food and water were autoclaved. The study was performed after obtaining the approval of the IACUC (Approval No: 2011-12-049). In order to determine tumor volume by a digital Vernier caliper, the greatest longitudinal diameter (length) and the greatest transverse diameter (width) were determined. Tumor volumes based on caliper measurements were calculated by the modified ellipsoidal formula: tumor volume=1/2(length × width^2^).

### Statistical analysis

The quantification of mRNA expression was performed with triplets (n≥3) and the data are presented as mean ± SEM. Statistically significant differences were estimated using P<0.01 and evaluated by one-way ANOVA. Statistical analyses were conducted using GraphPad Prism version 5 (GraphPad Software Inc., San Diego, CA).

## SUPPLEMENTARY MATERIALS FIGURES AND TABLES




