# Supplementary material for: Establishment and characterization of hypomethylating agent-resistant cell lines, MOLM/AZA-1 and MOLM/DEC-5
Source: Oncotarget. 2016 Dec 28;8(7):11748–62. doi: 10.18632/oncotarget.14342 (PMC5355301; doi:10.18632/oncotarget.14342)
Supplement: Supplementary file 1 [file oncotarget-08-11748-s001.pdf]

Establishment and characterization of hypomethylating agent-resistant cell lines, MOLM/AZA-1 and MOLM/DEC-5

SUPPLEMENTARY FIGURES AND TABLES

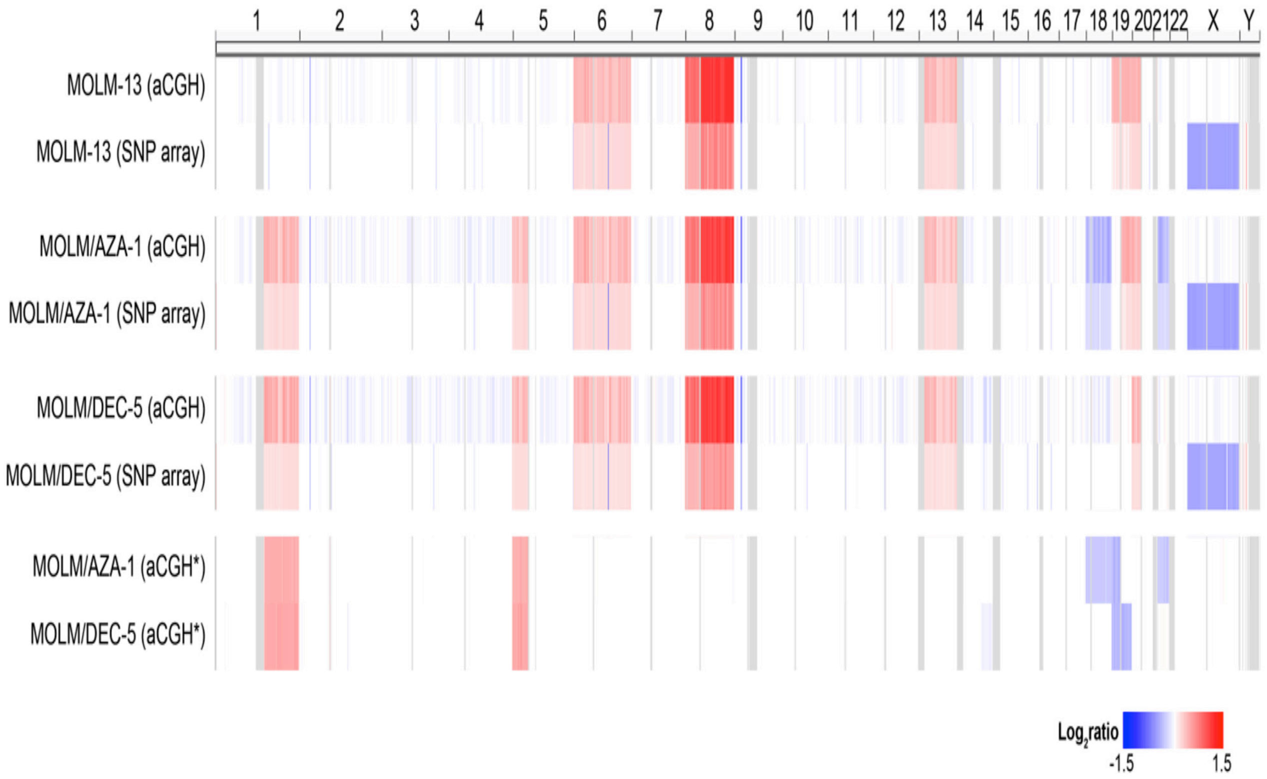

Supplementary Figure 1: HMA-resistance-specific CNAs. The numbers at the top represent individual chromosomes. The array-comparative genomic hybridization (aCGH) for MOLM/AZA-1 and MOLM/DEC-5 cell lines was performed with genomic DNA from the parental MOLM-13 cell line as a reference for each hybridization. All the CNAs identified in these aCGH analyses can be interpreted to be acquired during development of HMA resistance. Through the SNP microarray, all HMA-resistance-specific CNAs except one (14q24.2–q32.33 in MOLM/DEC-5) detected by aCGH were successfully validated. The upper three pairs show the results of aCGH and SNP array for MOLM-13, MOLM/AZA-1, and MOLM/DEC-5, respectively, while the lower pair shows HMA-resistance-specific CNAs.

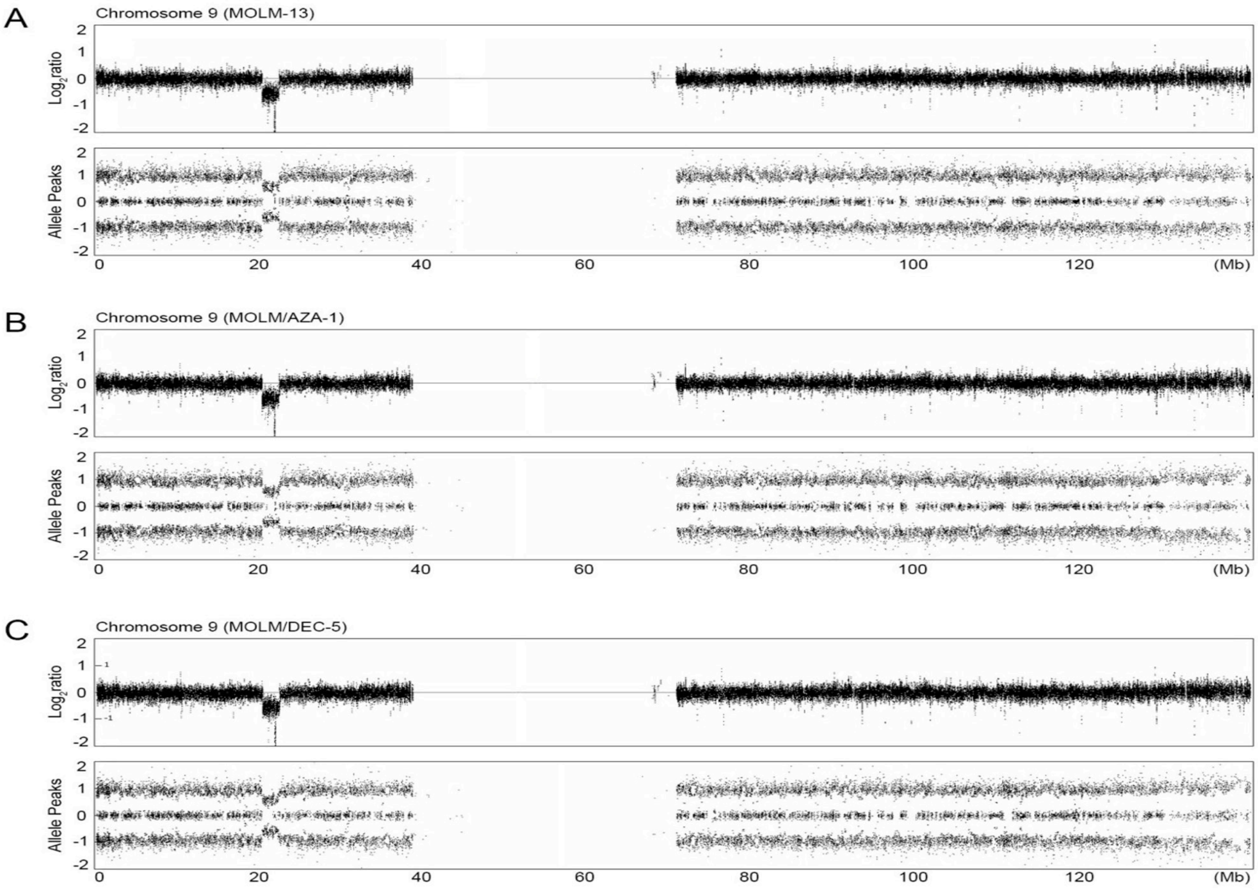

Supplementary Figure 2: Examples of copy number loss in a. MOLM-13, **b.** MOLM/AZA-1 and **c.** MOLM/DEC-5. MOLM-13 and HMA-resistant cell lines had a 9p21.3 deletion: a 1.5-Mb-sized region that encompasses the MLLT3 gene. Upper and lower panels represent copy number profiles and allele peak, respectively. The *x*-axis represents individual chromosomes and the *y*-axis represents signal intensity ratio on a $\log_2$ scale.

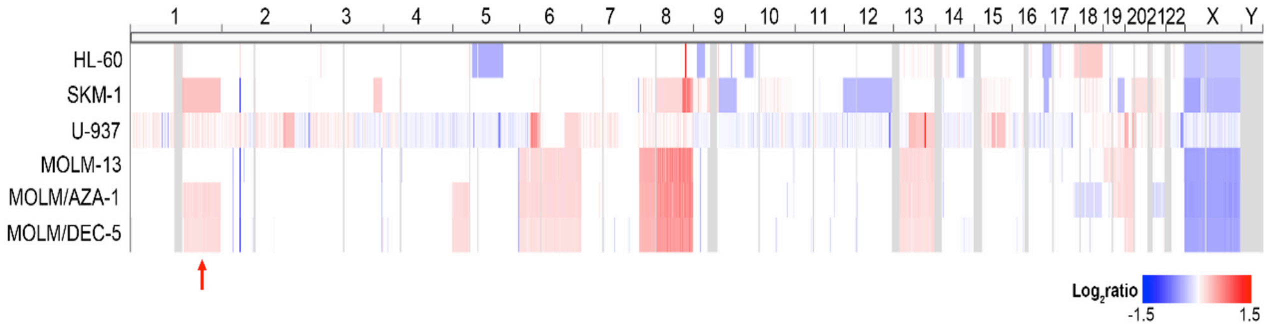

Supplementary Figure 3: Cell line specific CNAs. A HMA-resistance-specific CNA (red arrow) was identified in two myeloid neoplasm cell lines (SKM-1 and U-937). SNP microarray data of three AML cell lines (HL-60, SKM-1 and U-937) were obtained from Gene Expression Omnibus database (GSE36138). CNAs of MOLM-13 and two HMA resistance cell lines were identified using CytoScan® HD microarray data.

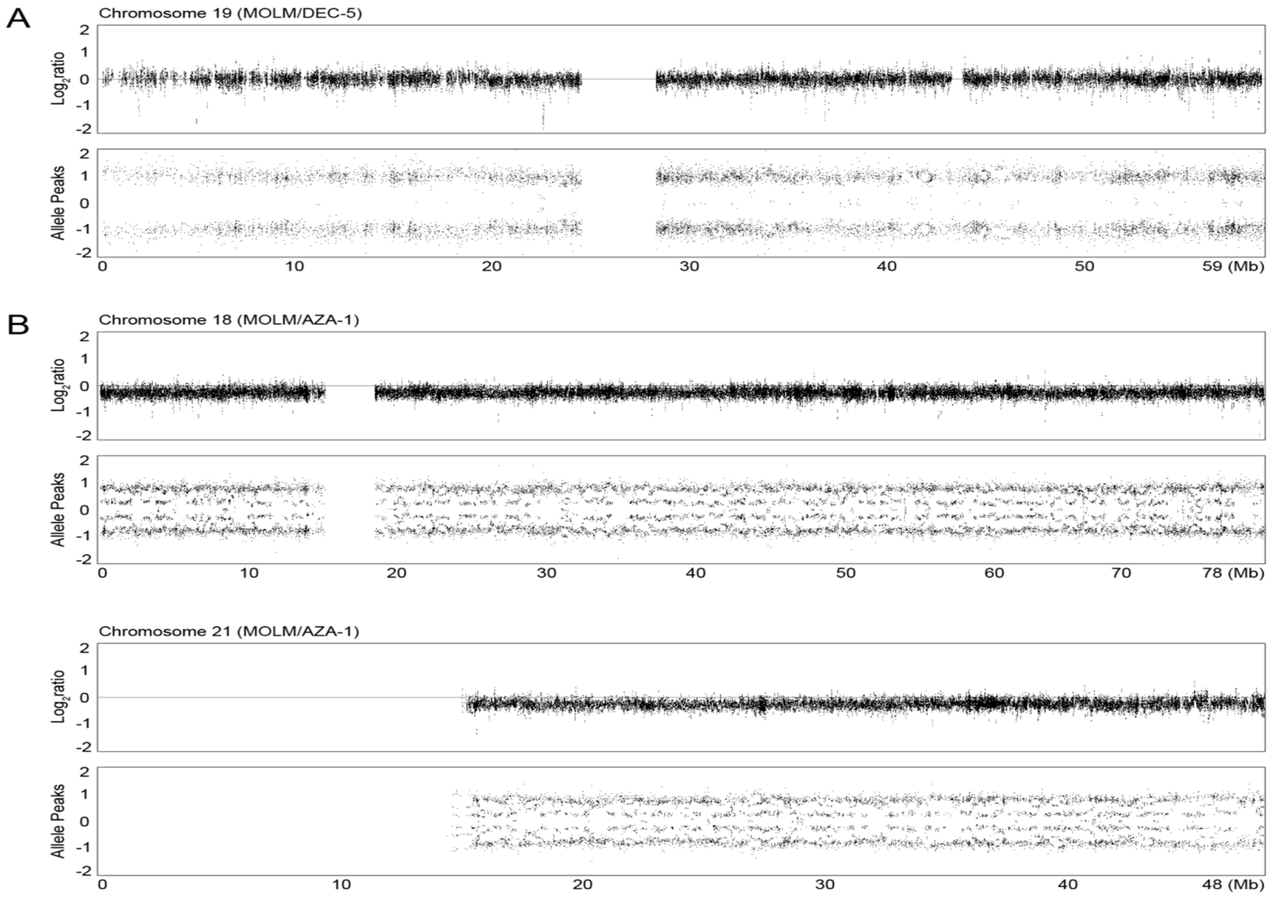

Supplementary Figure 4: Examples for log2 ratio of array-comparative genomic hybridization (array-CGH) and allele peak view of SNP microarray. Copy neutral LOH and mosaic loss event in HMA-resistant cell lines were identified. **a.** LOH in the 19p13.3–p12 region of the MOLM/DEC-5 genome; **b.** mosaic loss event in chromosome 18 and 21q of MOLM/AZA-1 genome.

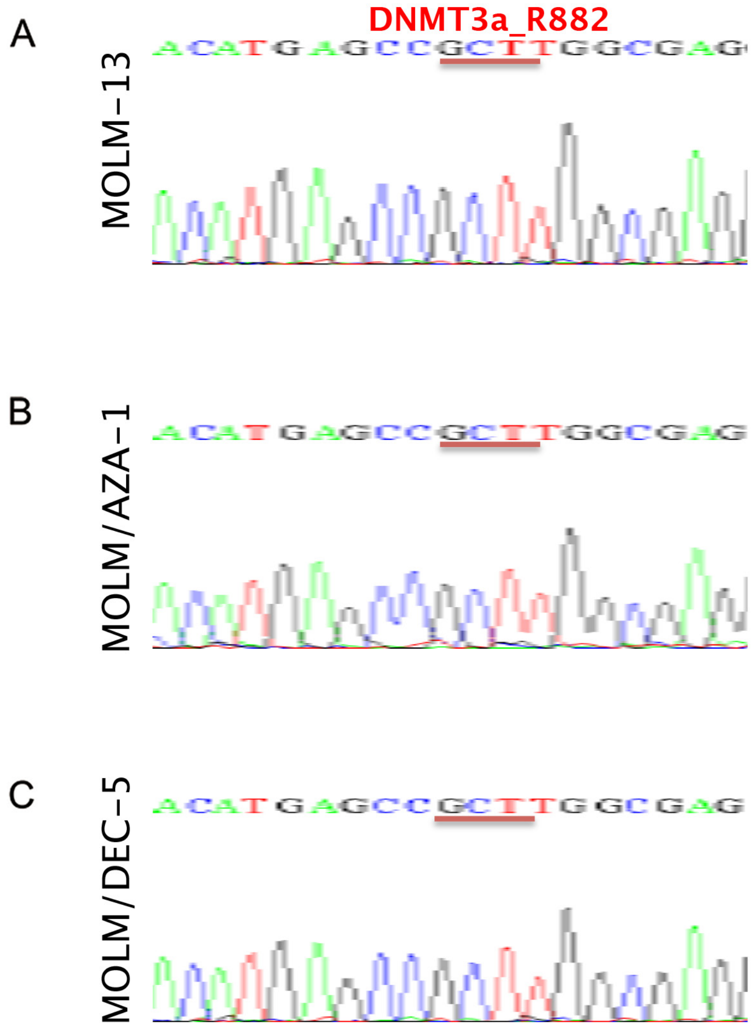

Supplementary Figure 5: Sanger sequencing of *codon R882*. a. MOLM-13, **b.** MOLM/AZA-1, **c.** MOLM/DEC-5 were indicated with wildtype sequence.

Supplementary Table 1: The short tandem repeat profiles of MOLM-13, MOLM/AZA-1, and MOLM/DEC-5 cell lines

	MOLM-13	MOLM/AZA-1	MOLM/DEC-5
D8S1179	13,14	13,14	13,14
D21S11	30,31	30,31	30,31
D7S820	10,12	10,12	10,12
CSF1PO	10,12	10,12	10,12
D3S1358	15,15	15,15	15,15
TH01	7,7	7,7	7,7
D13S317	10,11	10,11	10,11
D16S539	10,11	10,11	10,11
D2S1338	23,25	23,25	23,25
D1S433	12,14	12,14	12,14
vWA	16,17	16,17	16,17
TPOX	8,8	8,8	8,8
D18S51	13,15	14,15	14,15
Amelogenin	X, Y	X, Y	X, Y
D5S818	10,11	10,11	10,11
FGA	21,23	21,23	21,23

Supplementary Table 2: Pathway and ontology analysis of CNAs.

See Supplementary File 1

Supplementary Table 3: Primer Sequence

Gene	NM #	Forward	Reverse
MDR1	NM_000927	CAAGAAGCCCTGGACAAAG	GGACAGGCGGTGAGCAAT
SLC29A	NM_001078175	ACACCATCACCATGACAACC	CAGACCCAGCATGAAGAAGA
BCL9	NM_004326	CATCCATGCCAGGCCACAAC	GCAGCAGCTGGATTGCTCAT
ARNT	NM_001668	GGCCACAACTAGGTCCCACA	CAGGCTGAACCACCTGGGA
ABL2	NM_005158	GCTGTCCATCTCGCTCAGGT	CCCATCAGCCACTGTGGAGT
STK11	NM_000455	GACAGGTCCCAGAAGAGGAG	CCTCTGTGCCGTTCATACAC
TCF3	NM_003200	CAGACGAGGACGAGGACGAC	CCAGCTCCTTAAAGGCCTCGT
SMARCA4	NM_003072	TGGCCTGCAGTCCTACTATG	GACACCATTGACCATAAGCG
RUNX1	NM_001001890	AACAGAGACATTGCCAACCA	GTGATTTGCCCAGGAAAGTT
U2AF1	NM_006758	CGCCGTCGCAAGAAGCATAG	GATCTCTCGACCGCCTCCTG
DNMT1	NM_001130823	CCGGCCCCGGTTCTT	GGACCATGGAGCGCTTGA
DNMT3a	NM_022552	CGCTGAGCTCGTTTTGCA	GTAGATGGCTTTGCGGTACATG
DNMT3b	NM_001207055	CCTGCTGAATTACTCACGCCCC	GTCTGTGTAGTGCACAGGAAAA
β-actin	NM_001101	CCATCGTCCACCGCAAA	TCAAGAAAGGGTGTAACGCAACTA
