# Supplementary material for: Establishment and characterization of hypomethylating agent-resistant cell lines, MOLM/AZA-1 and MOLM/DEC-5
Source: Oncotarget. 2016 Dec 28;8(7):11748–62. doi: 10.18632/oncotarget.14342 (PMC5355301; doi:10.18632/oncotarget.14342)
Supplement: Supplementary file 2 [file oncotarget-08-11748-s002.docx]

**Supplementary Table 2.** Pathway and ontology analysis of CNAs

| **Category** | **Term** | **Count** | **P-value** | **Genes** |
| --- | --- | --- | --- | --- |
| KEGG | Transcriptional misregulation in cancer | 13 | 3.6x10^-11^ | SLC45A3, TMPRSS2, ERG, SS18, PRCC, LYL1, ELK4, NTRK1, MLLT1, H3F3A, PBX1, RUNX1, TCF3 |
| KEGG | Pathways in cancer | 11 | 4.9x10^-5^ | CCNE1, CBLC, BCL2, GNA11, NTRK1, TPR, RUNX1, TPM3, AKT2, ARNT, FH |
| GO_BP | B cell proliferation | 3 | 0.002 | BCL2, CD79A, IL7R |
| GO_BP | Positive regulation of transcription from RNA polymerase II promoter | 7 | 0.004 | SS18, FSTL3, BCL3, TCF3, ARNT, ERCC2, BCL9 |
| GO_BP | Cell differentiation | 4 | 0.004 | ERG, ELK4, ABL2, ARNT |
| GO_MF | Sequence-specific DNA binding | 5 | 0.007 | ERG, ELK4, BCL2, PBX1, ARNT |
| KEGG | Adrenergic signaling in cardiomyocytes | 5 | 0.009 | PPP2R1A, BCL2, TPM4, TPM3, AKT2 |
| GO_BP | B cell lineage commitment | 2 | 0.009 | BCL2, TCF3 |
| GO_MF | Chromatin binding | 5 | 0.010 | ERG, ELK4, BRD4, TPR, CIC |
| KEGG | PI3K-Akt signaling pathway | 7 | 0.013 | CCNE1, PPP2R1A, STK11, BCL2, JAK3, IL7R, AKT2 |
| KEGG | Thyroid cancer | 3 | 0.013 | NTRK1, TPR, TPM3 |
| GO_CC | Nucleus | 14 | 0.013 | ERG, STK11, ZNF521, CIC, ZNF331, CBLC, PRCC, ELK4, PBX1, MDM4, BRD4, TCF3, BCL9, AKT2 |
| GO_BP | Negative regulation of cell growth | 3 | 0.023 | STK11, BCL2, SMARCA4 |
| GO_CC | npBAF complex | 2 | 0.031 | SS18, SMARCA4 |
| GO_BP | DNA damage response, signal Transduction by p53 class mediator | 2 | 0.037 | BCL3, MDM4 |
| KEGG | Measles | 4 | 0.039 | CCNE1, FCGR2B, JAK3, AKT2 |
| GO_MF | Protein heterodimerization activity | 2 | 0.040 | BCL2, TCF3 |
| GO_BP | Positive regulation of transcription Elongation from RNA polymerase II promoter | 2 | 0.040 | ELL, BRD4 |
| KEGG | MicroRNAs in cancer | 4 | 0.043 | SLC45A3, CCNE1, BCL2, MDM4 |
| GO_CC | Transcription elongation factor complex | 2 | 0.043 | ELL, MLLT1 |
| KEGG | Signaling pathways regulating pluripotency of stem cells | 4 | 0.044 | LIFR, JAK3, TCF3, AKT2 |
| KEGG | Apoptosis | 3 | 0.044 | BCL2, NTRK1, AKT2 |
| GO_MF | Signal transducer activity | 3 | 0.047 | CBLC, GNA11, MALT1 |
| KEGG | Renal cell carcinoma | 3 | 0.057 | AKT2, ARNT, FH |
| KEGG | B cell receptor signaling pathway | 3 | 0.060 | MALT1, CD79A, AKT2 |
| GO_CC | Nuclear membrane | 3 | 0.061 | TFPT, BCL2, TPR |
| KEGG | Jak-STAT signaling pathway | 4 | 0.062 | LIFR, JAK3, IL7R, AKT2 |
| GO_BP | Tricarboxylic acid cycle | 2 | 0.065 | SDHC, FH |
| KEGG | Chronic myeloid leukemia | 3 | 0.066 | CBLC, RUNX1, AKT2 |
| KEGG | Tuberculosis | 4 | 0.076 | FCGR2B, BCL2, MALT1, AKT2 |
| GO_CC | RNA polymerase II transcription factor complex | 2 | 0.076 | TCF3, ARNT |
| GO_BP | B cell receptor signaling pathway | 2 | 0.076 | BCL2, CD79A |
| GO_BP | Homeostasis of number of cells within a tissue | 2 | 0.079 | BCL2, FH |
| KEGG | Small cell lung cancer | 3 | 0.084 | CCNE1, BCL2, AKT2 |
| KEGG | ErbB signaling pathway | 3 | 0.087 | CBLC, ABL2, AKT2 |
| GO_BP | Cell growth | 2 | 0.088 | BCL2, IL7R |
| KEGG | Prostate cancer | 3 | 0.089 | CCNE1, BCL2, AKT2 |
| GO_BP | Positive regulation of B cell proliferation | 2 | 0.091 | BCL2, TCF3 |
| GO_BP | Protein complex assembly | 2 | 0.091 | PPP2R1A, MDM4 |
| GO_BP | Spleen development | 2 | 0.094 | BCL2, BCL3 |
